# Why do paediatricians prescribe antibiotics? Results of an Italian regional project

**DOI:** 10.1186/1471-2431-9-69

**Published:** 2009-11-06

**Authors:** Maria Luisa Moro, Massimiliano Marchi, Carlo Gagliotti, Simona Di Mario, Davide Resi, the "Progetto Bambini a Antibiotici [ProBA]" Regional Group

**Affiliations:** 1 Agenzia Sanitaria e Sociale Regionale Emilia-Romagna, Area di Programma Rischio Infettivo, Viale Aldo Moro, 21; 40127 Bologna, Italy; 2 CeVEAS - AUSL Modena, Viale Muratori, 201; Modena, Italy

## Abstract

**Background:**

To investigate determinants of antibiotic prescription in paediatric care, as a first step of a multilevel intervention to improve prescribing for common respiratory tract infections (RTIs) in a northern Italian region with high antibiotic prescription rate.

**Methods:**

A two-step survey was performed: in phase I, knowledge, and attitudes were explored involving all family and hospital paediatricians of Emilia-Romagna and a sample of parents. In phase II, patient care practices were explored in a stratified random sample of visits, both in hospitals and family physician's clinics; parent expectations were investigated in a sub-sample of these visits.

**Results:**

Out of overall 4352 visits for suspected RTIs, in 38% of children an antibiotic was prescribed. Diagnostic uncertainty was perceived by paediatricians as the most frequent cause of inappropriate prescription (56% of 633 interviewed paediatricians); but, rapid antigen detecting tests was used in case of pharyngitis/pharyngotonsillitis by 36% and 21% of family and hospital paediatricians only. More than 50% of paediatricians affirmed to not adopt a "wait and see strategy" in acute otitis. The perceived parental expectation of antibiotics was not indicated by paediatricians as a crucial determinant of prescription, but this perception was the second factor most strongly associated to prescription (OR = 12.8; 95% CI 10.4 - 15.8), the first being the presence of othorrea. Regarding parents, the most important identified factors, potentially associated to overprescribing, were the lack of knowledge of RTIs and antibiotics (41% of 1029 parents indicated bacteria as a possible cause of common cold), and the propensity to seek medical care for trivial infections (48% of 4352 children accessing ambulatory practice presented only symptoms of common cold).

**Conclusion:**

A wide gap between perceived and real determinants of antibiotic prescription exists. This can promote antibiotic overuse. Inadequate parental knowledge can also induce inappropriate prescription. The value of this study is that it simultaneously explored determinants of antimicrobial prescribing in an entire region involving both professionals and parents.

## Background

Common respiratory tract infections (RTIs) are the most frequent cause of antibiotic prescriptions in paediatric outpatient care. Inappropriate use of antimicrobial agents for viral respiratory infections is common [1], leading to constant increase of bacterial respiratory pathogens [2]. Several determinants of inappropriate prescribing have been reported, including diagnostic uncertainty, lack of knowledge, socio-cultural and economic pressures, fear of litigation, meeting parental expectations [3], but their importance varies in different environments. Italy is one of the countries with the highest antibiotic consumption in Europe [4] as well as resistance rates [5], due to a number of factors, including opinions and traditions regarding how to treat infections, lack of a stringent national antibiotic policy, and characteristics of the antibiotic market [6]. In Emilia-Romagna, a northern Italian region of 4 million inhabitants, the regional prescription rate, at the community level, in 2005 was 1222 prescriptions/1000/year of systemic antibiotics in children aged 0-14, with amoxicillin associated with inhibitors of beta-lactamase, cephalosporins, macrolides and broad-spectrum penicillins being the most prescribed antibiotic classes (33%, 24%, 21% and 20% of all prescriptions, respectively) [7]. Data from the Regional surveillance system of antimicrobial resistance show high prevalence of erythromycin resistance in *Streptococcus pneumoniae *(40%) and *Streptococcus pyogenes *(21%) isolated from respiratory tract cultures of resident children in 2006, and medium prevalence level of penicillin non susceptible *Streptococcus pneumoniae *(PNSP) (6% of PNSP, high level resistance being around 2%) [7].

To improve antibiotic use by changing behaviour [8], a study was conducted aimed at identifying local determinants of antibiotic prescription for common RTIs which explored knowledge, attitudes, referred and actual practice of paediatricians and parents, in Emilia-Romagna. Family paediatricians (FPs) in Emilia-Romagna provide primary care to 79% of the overall paediatric population (children aged 0-14) and about 93% of children under age 6 years [9]. The present paper reports findings from this study, which represent the preliminary phase of the ProBA Project (Progetto Bambini e Antibiotici), a multilevel intervention aiming to reduce inappropriate antibiotic use in the paediatric population of Emilia-Romagna [10].

## Methods

### Study design

**Figure 1:**
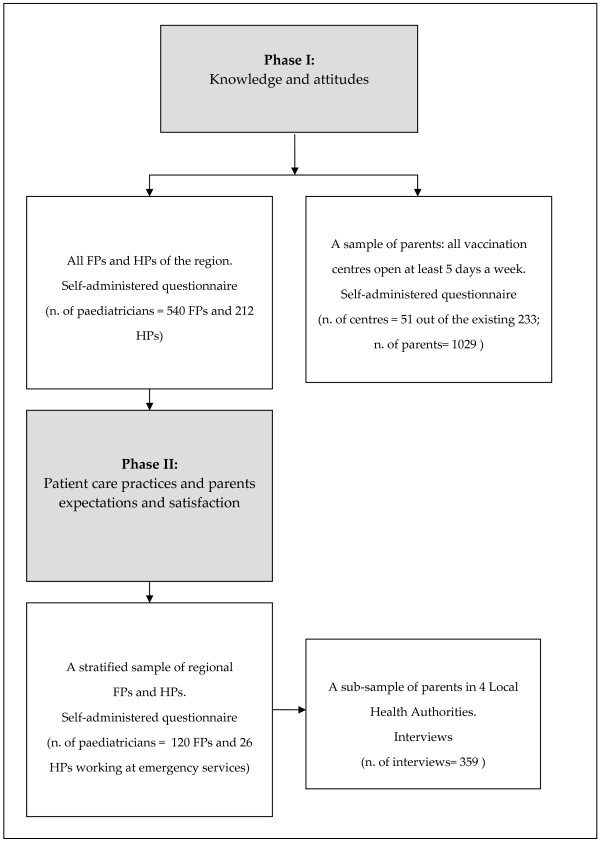
**Study design**.

A two-step knowledge, attitude and practice (KAP) survey was conducted, on the most common RTIs (upper RTIs and bronchitis/tracheitis). The first phase of the study aimed to explore, at regional level, knowledge and attitudes toward management of RTIs and antimicrobial resistance. All the FPs and hospital paediatricians (HPs) working in the Regional Health Service were included. Knowledge and attitude on the same topics were also investigated in a sample of parents. The second phase aimed to explore paediatrician actual practices of antibiotic prescription: the survey included a sample of children attending ambulatory or emergency department; parents' expectations and satisfaction were studied in a sub-sample of these consultations. Figure 1 summarizes the study design.

The survey on *Paediatricians' Knowledge and Attitudes *was conducted in September and October 2003, at the beginning of the RTIs "epidemic period". Two ad hoc questionnaires [9] were delivered to all FPs during CME events and to all HPs by mail. The questionnaires were not anonymous and included questions about general characteristics of the paediatrician, knowledge and opinions about causes of inappropriate antimicrobial prescribing, effective interventions for reducing it, and three clinical vignettes that explored the paediatricians' knowledge of common RTIs (Table 1). The questionnaire included closed questions with unordered answers as well as answers on a four points rating scale (from "strongly disagree" to "strongly agree"), which were grouped in the analysis as "disagree" or "agree".

**Table 1:** Clinical vignettes

	**Vignette description**	**Vignette objective**	**Correct answer**
**1**	▪ Evening visit at home ▪ 2 years of age ▪ 39°C for 24 hours, cough, seromucous nasal discharge ▪ negative rapid test	To measure the frequency of reported avoidance of antibiotic prescribing for viral URTI	No antibiotic prescription at first visit

**2**	▪ ambulatory visit ▪ 12 years of age ▪ pharyngitis, 38.7°C for 24 hours, no respiratory symptoms, large, red tonsils with purulent secretions	To measure the frequency of reported use of microbiological testing for *Streptococcus pyogenes *diagnosis	Given the high clinical score (according to McIsaac), Microbiological test before antibiotic prescription

**3**	▪ ambulatory visit ▪ 6 years of age ▪ 37.8°C, bilateral otalgia ▪ At examination bilateral otitis, erythema and bulging tympanic membrane	To measure the frequency of reported adoption of "wait and see strategy" (deferring antibacterial treatment for 48 to 72 hours)	It is reasonable to prescribe antibiotics, but it is better to defer antibacterial treatment for 48-72 hours

The survey on *Parental Knowledge and Attitudes *was conducted over ten days at the beginning of October 2003 at vaccination centres. Centres open at least 5 days per week were chosen based on opportunistic criteria (51 out of 233 centres existing in the region). A self-administered ad hoc anonymous questionnaire was offered to all parents [10]. Parents were excluded if not sufficiently proficient in Italian or if accessing the centre for reasons other than child vaccination. The questionnaire covered socio-demographic characteristics of parents and children, parental knowledge of bacteria, RTIs, antimicrobials and resistance and included closed questions with unordered answers as well as answers on a four points rating scale (from "strongly disagree" to "strongly agree").

*The survey on Paediatrician practices *was conducted during November and December 2003 on a sample of consecutive visits for suspected common RTI: a sample size of 4030 visits with a FP was calculated, according to a stratified (by local health authority- LHA) two stage sampling method and based on 31% expected inappropriate antibiotic prescription rate and 5% desired precision [11-14]. All 26 hospital emergency services were asked to describe at least 10-15 accesses for suspected RTI using a non anonymous questionnaire. In addition a random sample of 120 FPs stratified by LHAs was drawn; selected FPs were asked to complete a non anonymous ad hoc questionnaire for each suspected RTI visit, for a total of 35 questionnaires each. A sub-sample of 352 of these visits were also assessed to provide data for the *survey about Parental expectation/satisfaction*. Parents of children with RTI symptoms were interviewed by one of four interviewers opportunely trained: each interview had two phases. The first part of the interview, collecting data about parental expectation, was conducted in the ambulatory waiting room before the visit took place; the second part of the interview was conducted after completion of the visit and aimed to assess parental satisfaction. A unique code was used to link each parent's interview with the corresponding paediatrician's questionnaire. The questionnaire was anonymous and included closed questions with unordered answers as well as answers on a four points rating scale.

The study was not submitted for ethical approval, given the survey characteristics and the existing rules at the time the study was carried out; one of the ethics committee of the Regional Health Service confirmed that ethics approval was not necessary for the study.

### Statistical methods

Factors associated with antibiotic prescriptions were identified calculating Odds Ratios. The multivariate analysis was performed both by Logistic Regression and Generalized Estimated Equations (GEE) technique (the latter to assess clustering of children into the different paediatricians). GEE model produced results very similar to those obtained by the Logistic Regression model that were finally reported on this paper. All variables with a p-value below 0.2 in the univariate analysis were selected as candidates for inclusion in the model, along with other variables predefined (based on literature) as potential risk factors and/or confounders [15]. Significant improvement in log-likelihood function was the main criterion for keeping variables in the model. The effect of possible confounding variables was verified by introducing them in the final model and noting any changes in the coefficient of risk factors. Data were analyzed by SPSS software version 9.0.

## Results

### Paediatricians' knowledge and attitude

Response rate was 84% for FPs (453 respondents) and 85% for HPs (180 respondents). Age (mean 48 years) and time since completing specialization (about 17 years) were similar in both paediatrician categories. The proportion of women was 64% among FPs and 55% among HPs. Regarding office organization, 30% of FPs worked in association with other paediatricians; 18% had a secretary or nurse helping with ambulatory activities; 57% used software to manage patient data. The perceived causes of inappropriate antibiotic prescription most frequently reported were diagnostic uncertainty (54% of FPs and 60% of HPs) and parental expectations of a prescription (23% of FPs and 13% of HPs). The HPs also frequently mentioned difficulty in assuring follow-up (53%). FPs indicated bronchitis as the RTI most frequently causing diagnostic uncertainty (43%), while HPs pointed to otitis (36%). Among measures for reducing inappropriate antibiotic prescription, the most commonly were: availability of rapid diagnostic tests (rapid antigen detection test -RADT- for diagnosing group A beta-hemolytic streptococcus and/or urinary dipstick tests for diagnosis of urinary tract infections) (54% FPs, 61% HPs), development/implementation of specific guidelines (44% FPs, 57% HPs), provision of independent information (46% FPs, 41% HPs) and implementation of measures aimed at involving families (35% FPs, 34% HPs).

Thirty five percent of FPs and 64% of HPs correctly answered the first vignette; 81% and 88% correctly answered the second; 50% and 35% the third (see Table 1). There was significant variability in the proportion of correct answers among the 11 LHAs: from 29% to 63% for the first vignette, from 69% to 92% for the second, from 19% to 56% for the third.

### Parental knowledge and attitude

A total of 1029 questionnaires were completed. Parental mean age was 35 years for mothers and 37 years for fathers. Education level (referring to at least one parent) was primary school diploma (30%), secondary school diploma (50%) and university degree (21%). Both parents were employed in 80% of cases, and only one in the other 20%. At least one parent was born abroad in 11% of cases. Children were under three years of age in 71% and had no siblings in 48% of cases. Persons most frequently caring for a sick child were the mother (67%), the grandmother/grandfather (47%) and/or the father (15%). In 41% of questionnaires, bacteria were indicated as possible cause of common cold; 47% of parents believed that in most cases RTI would resolve spontaneously without the help of a doctor. Parents indicated that, beside bacteria, viruses also can be the cause of otitis, sore throat and cough in 27%, 39% and 35% of cases, respectively. Thirty seven percent of parents considered antibiotics efficacious against viruses; 35% of parents thought that paediatricians sometime prescribe unneeded antibiotics.

### Paediatrician practices

Overall, 4352 visits for suspected common RTI were recorded during the study period: 4052 by FPs and 300 by HPs. The symptom most frequently recorded was cough (70%), followed by rhinorrhea (63%) and sore throat (52%). Nearly half of the children presented only symptoms of common cold (48%). Fever and otalgia were more frequent among children accessing the hospital than those at outpatient clinics (51% *vs *37% and 29% *vs *19%, respectively). The most frequent diagnosis was rhinopharyngitis (34%), followed by pharyngitis/pharyngotonsillitis (23.7%) and otitis (13.6%). In 1242 cases (29%), the paediatrician could not distinguish viral or bacterial etiology. Otoscopy was performed in 83% of the visits by FPs and 90% by HPs; in case of pharyngitis or pharyngotonsillitis, 36.1% of FPs and 21.0% of HPs, respectively, performed a microbiological test (RADT or culture).

Antimicrobials were prescribed in 1645 cases (37.8%), most frequently in cases of bronchitis or otitis (69% of children with one of these diagnoses), and pharyngotonsillitis (59%). Variables significantly associated with antibiotic prescription were: parental expectation of antibiotic prescription (as perceived by the paediatrician); a parent born abroad; tonsillar exudate or redness; facial pain; otalgia; absence of diarrhoea; cervical adenopathy; bulging tympanic membrane; otorrhea; absence of rhinorrhea; fever > 38°C (Table 2). No difference in the probability of antibiotic prescription was found between ambulatory practices and hospital emergency service paediatricians (OR = 1.0, 95% CI 0.74-1.36, for type of physician entered in the previous model). The presence of an interviewer in the ambulatory was negatively associated with antibiotic prescription (OR = 0.59, 95% CI 0.42-0.83 in the previous model limited to FPs only).

**Table 2:** Factors associated with antibiotic prescription: visits for common respiratory infections. Univariate and multivariate analysis

**Variable**	N* of children	**Antibiotic prescriptions** **%**	**OR** **(95% CI)**	**Adjusted OR** **(95% CI)**
Child's age				
≤2 years	1370	33%	1	1
>2 years	2943	40%	1.35 (1.18 - 1.55) *	0.98 (0.80 - 1.19)
Parent born abroad				
No	625	37%	1	1
Yes	3653	45%	1.37 (1.16 - 1.63)*	1.71 (1.36 - 2.15) *
Day care attendance				
No	1461	34%	1	1
Yes	2683	39%	1.27 (1.11 - 1.45)*	1.12 (0.93 - 1.35)
Was the parent expecting an antibiotic prescription?				
No	2524	19%	1	1
Yes	1050	77%	14.21 (11.94 - 16.91)*	12.83 (10.43 - 15.79)*
Don't know	712	49%	4.15 (3.48 - 4.96)*	3.48 (2.82 - 4.30) *
Exudate or tonsillar hyperemia				
No	2077	31%	1	1
Yes	2275	44%	1.70 (1.50 - 1.93)*	1.63 (1.36 - 1.96) *
Facial pain				
No	4284	37%	1	1
Yes	68	71%	4.03 (2.39 - 6.82)*	4.85 (2.25 - 10.46) *
Otalgia				
No	3505	33%	1	1
Yes	847	60%	2.79 (2.39 - 3.25)*	2.02 (1.60 - 2.55) *
Periorbital edema				
No	4284	38%	1	1
Yes	68	47%	1.47 (0.91 - 2.38)	1.67 (0.89 - 3.14)
Diarrhoea				
No	4224	38%	1	1
Yes	128	24%	0.52 (0.34 - 0.78)*	0.55 (0.32 - 0.93) *
Cervical adenopathy				
No	3457	33%	1	1
Yes	895	57%	2.67 (2.30 - 3.11)*	2.09 (1.70 - 2.59) *
Bulging tympanic membrane				
No	3959	34%	1	1
Yes	393	78%	6.77 (5.29 - 8.65)*	5.68 (4.06 - 7.94) *
Otorrhea				
No	4281	37%	1	1
Yes	71	96%	(12.20 - 123.60)*	28.06 (6.32 - 124.51)*
Rhinorrhea				
No	2.521	40%	1	1
Yes	1.831	35%	0.83 (0.73 - 0.94)*	0.72 (0.61 - 0.86) *
Fever				
≤38°C	2713	29%	1	1
>38°C	1639	53%	2.86 (2.52 - 3.25) *	2.34 (1.97 - 2.79) *
Associated chronic diseases				
No	4135	38%	1	1
Yes	217	44%	1.30 (0.98 - 1.71)	1.29 (0.88 - 1.88)
Physician age**				1.02 (0.95-1.10)

* statistically significant p < 0.05

**OR for 5 years of age increase

### Parental expectation/satisfaction

Three hundred and fifty nine parents were interviewed during a consultation for suspected RTI: in the pre-visit phase of the interview, 62 parents (17%) declared to be expecting an antibiotic prescription. Variables significantly associated with this expectation were previous antibiotic prescription (OR = 2.26, 95% CI 1-5.07), sore throat (OR 2.57, 95% CI 1.21-5.48) and ear pain (OR 2.73, 95% CI 1.09-6.83). In a large proportion of cases, parental expectations of antibiotic prescription were not correctly perceived by paediatricians: in 24% of cases, the paediatricians believed parents wanted an antibiotic when they actually did not; in 10.5% of cases, the paediatricians did not perceive the parents' expectation of an antibiotic. A multivariate model including only the 359 consultations where parents were interviewed, showed an association between parents' actual expectations of an antibiotic and antibiotic prescription ("Do you think your child needs an antibiotic today" yes: OR 9.87, 95% CI 3.09-31.45; I don't know OR 3.99, 95% CI 1.60-9.97), while the Odds Ratios and probability values of the association of all the other variables were similar to those observed in the full population model, including the association with parental expectation of antibiotics as perceived by the paediatrician. In the post-visit phase of the survey, 32.5% of parents stated that they received an antibiotic prescription; irrespective of the prescription received, almost all parents were satisfied (98% of those with an antibiotic prescription and 99.5% of those without). All the parents declared that they would comply with the paediatrician's prescription.

## Discussion

This study confirms that the high volume of antibiotic prescription in the paediatric population observed in the region is due, at least partly, to inappropriate prescribing: 38% of all children referred for suspected RTI and 22% of those with RTI diagnosis likely due to viral causes (bronchitis, laryngotracheitis, common cold) received an antibiotic prescription. This figure is higher than that reported by similar ad hoc studies for the same type of RTIs [16]. In addition, paediatricians' attitudes tend to be biased towards antibiotic overuse, as confirmed by the clinical vignette answers. Considerable variation in clinical decisions referred for the three clinical vignettes was observed among paediatricians in different LHAs, indicating that factors different from the child's clinical condition influence the likelihood of antibiotic prescribing [3].

The study also confirms that in Emilia-Romagna determinants of antibiotic prescriptions are complex, not evenly distributed, and not clearly perceived and understood by all the actors in the parent-doctor-child relation.

Regarding physicians, two principal determinants of overprescribing were identified: diagnostic uncertainty and perceived parental expectations of an antibiotic prescription.

Diagnostic uncertainty, which has been repeatedly recognized as one of the determinants for antibiotic overuse in RTIs [3], is perceived by paediatricians in this study as a crucial determinant of inappropriate antibiotic prescription. However, a wide gap exists between what is referred and actual practice: a high proportion of health professionals suggest using a rapid test as a possible solution to reduce diagnostic uncertainty for pharyngotonsillitis, both as an opinion and in clinical vignettes. In clinical practice, a much smaller proportion of health professionals use RADT or culture when needed. This can be partially explained by time constraints due to office organization and possibly by the financial costs that the test entails [17].

Parental expectations of receiving an antibiotic is perceived by a limited proportion of paediatricians as a reason for inappropriate prescription. In observed practice, on the contrary, paediatricians' perception that parents expect antibiotics is the second factor most strongly associated with prescription, the first being the presence of otorrhea. In addition, paediatricians tend to misinterpret parental expectations: when parents are directly interviewed, one in four parents that did not expect an antibiotic received it, while one in ten that expected an antibiotic did not actually receive it. This confirms the result of several other studies [18-22].

The lack of communication with parents can be partially explained by suboptimal general ambulatory organization that could act as a barrier to improvements in clinical practice: few paediatricians work in association and even fewer have any help in organising ambulatory activities. Organisational changes would, therefore, save time for longer consultations and possibly improve communication with parents.

This study also confirms that specific individual parental characteristics may influence prescribing: having one non Italian parent is significantly associated with receiving antibiotic prescription (OR 1.7). Several other studies have convincingly shown that the socioeconomic status of parents, such as having a high household income [23], or being a doctor or pharmacist [24], may influence antibiotic prescribing. This is because these subgroups may be better informed about appropriate antibiotic therapy or have greater flexibility in taking time off work to bring their children back to a physician if symptoms do not resolve [23].

Regarding parents, two principal determinants of potential overprescribing were identified: not being sufficiently informed about the fact that "not all bugs need drugs" [25] and the natural history of common RTIs, and the lack of any support in managing a sick child.

The parents interviewed were largely comparable to the general population of parents living in Emilia-Romagna [26], the only significant difference being the smaller percentage of parents born abroad due to selection criteria. In general, parents interviewed were moderately to well educated, had one child and, in a large percentage, both parents are employed. The results of the survey regarding parental knowledge and attitudes are somewhat composite: almost half of the parents knew that most RTIs would resolve spontaneously, but a large proportion believed that rhinitis is due to bacteria and that antibiotics are needed against viruses. It seems that the distinction between bacterial and viral infections, their natural history, signs and symptoms is not clear. This is supported also by the observation that as much as 48% of children accessing ambulatory practice present exclusively with rhinitis symptoms.

All the above results confirm the crucial role of cultural factors as well as social factors in determining the pattern of antimicrobial prescribing in a region, as pointed out by Harbarth [27].

The ProBA survey results, based on a multistep design, provide several insights to understanding determinants of antibiotic prescription in paediatric care. Comparison of answers relating to knowledge, attitude, referred behaviour and actually observed practices illustrates the complex web of determinants better than a single source of information and represents one of the strengths of this study, along with the fact that both parents' and paediatricians' determinants were explored and all paediatricians of the region, not just a sample, were included in the survey.

Moreover, given the high response rate, survey results relative to health professionals can be considered representative of the regional situation.

On the other hand, the study presents some limitations: the presence of the Hawthorne effect is likely in the interviews, as indicated by the association between the presence of interviewers in the ambulatory and reduced probability of receiving an antibiotic prescription; comparison between different sources and data collection techniques should however increase data reliability [28]. Another potential limit of the study is the incomplete control over potential selection of both the children and parents' population: however, the enrolment of the requested number of children was achieved by all paediatricians in due time, making selection unlikely. Finally the decision to exclude from the survey parents not sufficiently proficient in Italian prevents elaboration of knowledge and attitudes of the foreign-born population.

## Conclusion

This study shows a complex situation that should be managed with multi-level interventions involving both parents and paediatricians. Use of rapid tests to reduce diagnostic uncertainty should be expanded; clear and feasible recommendations for the management of common RTIs in children should be implemented, information tools designed for parents should be developed and disseminated. Action aimed at facing these aspects is planned in the second phase of the ProBA project. But other changes should be promoted: ambulatory organization should be improved, professionals' communication skills should be developed, community-wide activities should be promoted to improve correct recourse to antibiotics. Finally, work places should be flexible enough to allow parents to assist their children during illness.

## Competing interests

The authors declare that they have no competing interests.

## Authors' contributions

MLM conceived the study, participated in its design and supervision; and in the drafting of the manuscript. MM: participated in the design of the study and performed the statistical analysis. CG: conceived the study, participated in its design and in the drafting of the manuscript. SDM: participated in the analysis and interpretation of data; drafting of the manuscript. DR: Conception and design; and drafting of the manuscript.

All authors read and approved the final manuscript.

## Pre-publication history

The pre-publication history for this paper can be accessed here:


